# ALDH2 Inhibition Potentiates High Glucose Stress-Induced Injury in Cultured Cardiomyocytes

**DOI:** 10.1155/2016/1390861

**Published:** 2016-11-02

**Authors:** Guodong Pan, Mandar Deshpande, Rajarajan A. Thandavarayan, Suresh Selvaraj Palaniyandi

**Affiliations:** ^1^Division of Hypertension and Vascular Research, Department of Internal Medicine, Henry Ford Health System, Detroit, MI 48202, USA; ^2^Department of Cardiovascular Sciences, Center for Cardiovascular Regeneration, Houston Methodist Research Institute, Houston, TX, USA; ^3^Department of Physiology, Wayne State University, Detroit, MI 48202, USA

## Abstract

Aldehyde dehydrogenase (ALDH) gene superfamily consists of 19 isozymes. They are present in various organs and involved in metabolizing aldehydes that are biologically generated. For instance, ALDH2, a cardiac mitochondrial ALDH isozyme, is known to detoxify 4-hydroxy-2-nonenal, a reactive aldehyde produced upon lipid peroxidation in diabetic conditions. We hypothesized that inhibition of ALDH leads to the accumulation of unmetabolized 4HNE and consequently exacerbates injury in cells subjected to high glucose stress. H9C2 cardiomyocyte cell lines were pretreated with 10 *μ*M disulfiram (DSF), an inhibitor of ALDH2 or vehicle (DMSO) for 2 hours, and then subjected to high glucose stress {33 mM D-glucose (HG) or 33 mM D-mannitol as an osmotic control (Ctrl)} for 24 hrs. The decrease in ALDH2 activity with DSF pretreatment was higher in HG group when compared to Ctrl group. Increased 4HNE adduct formation with DSF pretreatment was higher in HG group compared to Ctrl group. Pretreatment with DSF leads to potentiated HG-induced cell death in cultured H9C2 cardiomyocytes by lowering mitochondrial membrane potential. Our results indicate that ALDH2 activity is important in preventing high glucose induced cellular dysfunction.

## 1. Introduction

ALDH is a family of multiple homotetrameric enzymes [1]. As the name implies, ALDH enzymes metabolize aldehydes into carboxylic acids. These aldehydes include aliphatic and aromatic aldehydes from the environment, food, and those generated during cell metabolism. ALDH plays a major role in detoxifying reactive aldehydes. When ALDH2 was overexpressed, the deleterious effects of acetaldehyde in the heart were diminished [2]. A single point mutation in ALDH2 greatly reduces ALDH2 activity in East Asians, which leads to accumulation of acetaldehyde and ultimately facial flushing [3] and other harmful effects [4]. Moreover, this mutation is reportedly associated with diabetic complications [5] and coronary diseases in East Asians. It is suggested that the toxicity of reactive aldehydes plays main role in the pathogenesis.

Hyperglycemia-induced oxidative stress is implicated in the pathogenesis of diabetic complications [6]. We focus on the secondary products of oxidative stress, that is, reactive aldehydes. Toxic reactive aldehydes such as 4-hydroxy-2-nonenal (4HNE) are produced by lipid peroxidation during oxidative stress in the mitochondria [7]. 4HNE is especially toxic as it forms adducts with proteins and leads to cellular dysfunction [7–14].

In another recent report, we have shown that pharmacological inhibition of ALDH2* per se* induces mitochondrial dysfunction and cell death [15]. To provide an extension of our previous results, we planned to investigate the effect of ALDH2 inhibition using disulfiram (DSF), an ALDH2 inhibitor, on high glucose stress in H9C2 cardiomyocyte cell lines.

## 2. Materials and Methods

### 2.1. H9C2 Cell Culture and Treatment Protocol

H9C2 cell lines {ATCC CLR-1446, routinely used as cardiomyocytes [16–18]}, were cultured and grown in DMEM medium. The cells were grown to reach 60–70% confluence and subsequently used in the experiments.

After 1 hr of starving, H9C2 cells were treated with DSF (Santa Cruz Chemicals, Santa Cruz, CA), an inhibitor of ALDH2 or DMSO for 2 hours, and then subjected to high glucose stress for 24 hours. The high glucose stress was induced by replacing the culture medium with 33 mM of D-glucose (HG). We maintained the glucose concentration in the cell culture medium throughout the study period by measuring the glucose level using a glucometer. To nullify the osmotic stress caused by high concentration of solute in the culture medium is not the cause for the observed cellular stress, the culture medium of another set of cells was replaced with the same 33 mM D-mannitol (Ctrl).

We chose 2 doses for DSF, that is, 10 *μ*M and 2 *μ*M after performing a dose-response curve using DSF in inhibiting ALDH2 activity as shown in Supplementary Figure  1 (see Supplementary Material available online at http://dx.doi.org/10.1155/2016/1390861). We used 2 *μ*M for the mitochondrial studies as mitochondrial oxygen consumption rate (OCR) was decreased below detection level with 10 *μ*M DSF treatment.

### 2.2. Intracellular ROS Measurement

Intracellular ROS can oxidize the nonfluorescent 29,79-dichlorofluorescein diacetate (DCFH-DA) to generate fluorescent 29,79-dichlorofluorescein (DCF). Fluorescence intensity of DCF can be measured using a fluorimeter. Therefore to measure intracellular ROS in H9C2 cells, we used the DCF kit and followed the manufacturer's instructions (Abcam Inc., Cambridge, MA). Briefly, cells were washed twice with PBS after the study protocol, and then the ROS detection solution was added. Cells were stained at 37°C in the dark for 20 min. The fluorescence intensity of DCF was measured using a fluorimeter at 504 nm excitation and 524 nm emission wavelengths.

### 2.3. ALDH Activity Assay

ALDH2 activity was measured by following the procedure described elsewhere [19, 20]. In brief, enzymatic activity of ALDH2 from cell lysate was determined spectrophotometrically by using the reductive reaction of NAD+ to NADH at *λ*340 nm. All assays were carried out at 25°C in 0.1 M sodium pyrophosphate buffer, pH = 9.5 with 2.4 mM NAD+ as a cofactor and 10 mM acetaldehyde as the substrate.

### 2.4. Western Blot Analysis of 4HNE Protein Adducts

4HNE protein adducts were evaluated by Western immunoblot as described earlier [21, 22]. In brief, protein samples from cell lysates were separated using SDS-PAGE and the proteins were then transferred to immobilon-P membranes (Millipore, Billerica, MA). Anti-4HNE-Cys/His/Lys rabbit antibody (Millipore) and anti-*β*-actin mouse monoclonal antibody (SCBT, Santa Cruz, CA) at a concentration of 1 : 1000 (4°C overnight) were used. The bound antibody was visualized with horseradish peroxidase- (HRP-) coupled secondary antibody and chemiluminescence detection reagents.

Intensity of scanned Western blot images was analyzed using Image J software. The ratios of levels of 4HNE protein adducts and *β*-actin, the loading control, were calculated and plotted as graphs.

### 2.5. Trypan Blue Exclusion Cell Death Assay in H9C2 Cardiomyocytes

Cells grown at 60–70% confluence were starved for 1 hr using DMEM media without serum. The plates were then divided into the following treatment groups (*n* = 6 plates each). At the end of the protocol, cells were washed with TBS buffer and trypsinized. Trypan blue was added at a concentration of 1 : 1 and subsequently an automated cell counter (Bio-Rad) was used to determine % of live cells remaining.

### 2.6. Mitochondrial ROS Measurement

MitoSOX reagent was used to measure mitochondrial ROS as explained in the manufacturer's instructions. 1.0–2.0 mL of 5 *μ*M MitoSOX reagent working solution was used to cover H9c2 cells and incubated for 10 minutes at 37°C, protected from light. After washing cells with warm buffer, we processed the cells for imaging using florescent microscope with absorption at 510 nm and emission 580 nm. We also quantified the fluorescence using Synergy H1 Multi-Mode Reader (BioTek Inc.).

### 2.7. Measurement of Mitochondrial Transmembrane Potential

5,5′,6,6′-Tetrachloro-1,1′,3,3′-tetraethylbenzimidazolyl-carbocyanine iodide (JC-1; Sigma-Aldrich) was used to determine changes in mitochondrial transmembrane potential (ΔΨ_m_). JC-1 (10 *μ*L; 200 *μ*M; final concentration 2 *μ*M) was added to H9c2 cells on coverslips, incubated for 45 min in the dark, and washed twice with PBS. JC-1 fluorescence was measured from a single excitation wavelength (490 nm) with dual emission (shift from green at 530 nm to red at 590 nm) using a fluorimeter. Loss of mitochondrial ΔΨ_m_ is presented as the relative ratio of green to red fluorescence.

### 2.8. Measurement of Oxygen Consumption Rate (OCR) in H9C2 Cardiomyocytes

H9C2 cells were seeded at 30,000 cells/well onto Bioscience V7 culture plates in growth medium containing 15% FBS (fetal bovine serum) on the first day. The next day, medium was replaced, and cells were grown in the culture growth medium. Within 24 hrs of seeding, a confluent monolayer of H9C2 cells was formed and then cells were used as described below. XF24 Analyzer (Seahorse Biosciences) was used to measure OCR to be monitored in real time function in H9C2 cardiomyocytes as mentioned in our earlier report. For all measurements, the culture medium was changed to serum-free media for 1 hr prior to assay. At the end of the treatment, cells were washed with buffer and Cell Mito Stress Test (CMST) media for cell bioenergetics measurements were added into each well.

The plate was then transferred to the XF24 instrument and the experiment was initiated. Oligomycin, an ATP synthase inhibitor (to check the ATP mediated respiration); FCCP, a known uncoupler and proton translocating ionophore (to determine maximal respiration and the mitochondrial reserve capacity); and antimycin A, an inhibitor of the mitochondrial electron transport chain (to check OCR from nonmitochondrial respiration related) prepared in CMST were injected sequentially through ports in the Seahorse Flux Pak cartridges and then mitochondrial OCR was recorded.

### 2.9. Statistical Analysis

Data is presented as mean ± standard error of the mean (SEM). One-way ANOVA was used to compare the groups. Statistical significance was achieved when *p* was <0.05.

## 3. Results

### 3.1. Effect of DSF Pretreatment on ROS Levels in Cultured H9C2 Cardiomyocytes Subjected to High Glucose Stress

**Figure 1:**
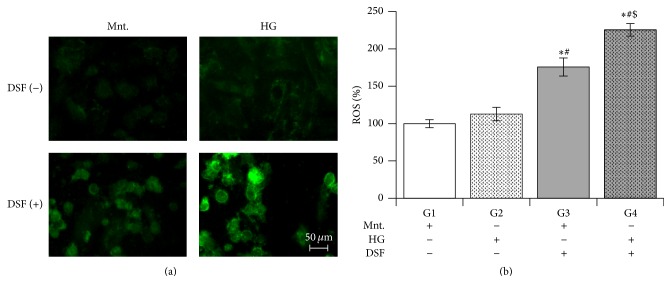
ALDH2 inhibition by pretreating with disulfiram (DSF) and reactive oxygen species (ROS) levels. (a) Representative photomicrographs of DCF stained cells from respective treatments. (b) Increase in ROS in cultured H9C2 cardiomyocytes subjected to high glucose stress (G2) compared to equimolar mannitol (G1). Disulfiram (DSF) pretreatment increased ROS levels in both mannitol (G3) and high glucose (G4) groups. The data expressed are mean ± SEM. *N* = 4–6. ^*∗*^ *p* < 0.05 versus G1, ^#^ *p* < 0.05 versus G2, and ^$^ *p* < 0.05 versus G3.

DSF pretreatment significantly increased ROS levels in cultured H9C2 cardiomyocytes subjected to high glucose stress compared to DSF-untreated cells presented with equimolar concentrations of glucose or mannitol. Surprisingly, DSF pretreatment significantly increased ROS levels in the mannitol group compared to the DSF-untreated group. However, DSF pretreatment enhanced the ROS increase significantly higher in H9C2 cells with high glucose stress compared to cells in the control (mannitol) group (Figure 1).

### 3.2. Effect of DSF Pretreatment on ALDH2 Activity in Cultured H9C2 Cardiomyocytes Subjected to High Glucose Stress

DSF pretreatment significantly reduced ALDH2 activity in cultured H9C2 cardiomyocytes subjected to high glucose stress compared to DSF-untreated cells presented with equimolar concentrations of glucose or mannitol.

**Figure 2:**
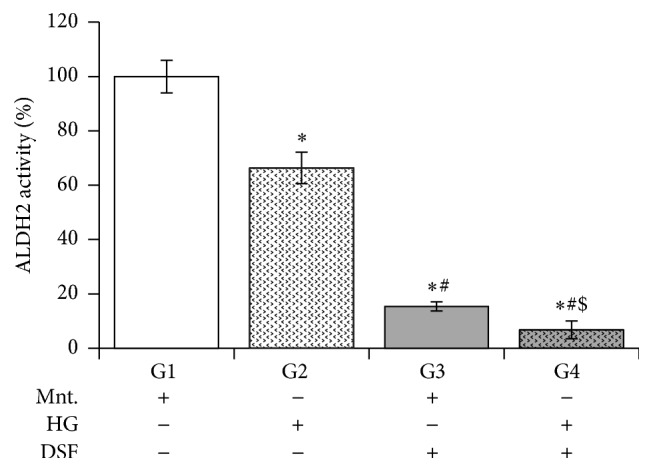
Disulfiram (DSF) pretreatment and ALDH2 activity. DSF 10 *μ*M reduces ALDH2 activity in both HG and Ctrl groups. The data expressed are mean ± SEM. *N* = 4–6. G1, G2, G3, and G4 depict mannitol, high glucose stress, DSF + mannitol, and DSF + high glucose stress. G1, G2, G3, and G4 depict mannitol, high glucose stress, DSF + mannitol, and DSF + high glucose stress, respectively. ^*∗*^ *p* < 0.05 versus G1, ^#^ *p* < 0.05 versus G2, and ^$^ *p* < 0.05 versus G3.

Furthermore, this DSF pretreatment significantly reduced ALDH2 activity in H9C2 cells with high glucose stress compared to cells in the control (mannitol) group (Figure 2).

### 3.3. Effect of DSF Treatment on 4HNE Adducts Formation in Cultured H9C2 Cardiomyocytes Subjected to High Glucose Stress

**Figure 3:**
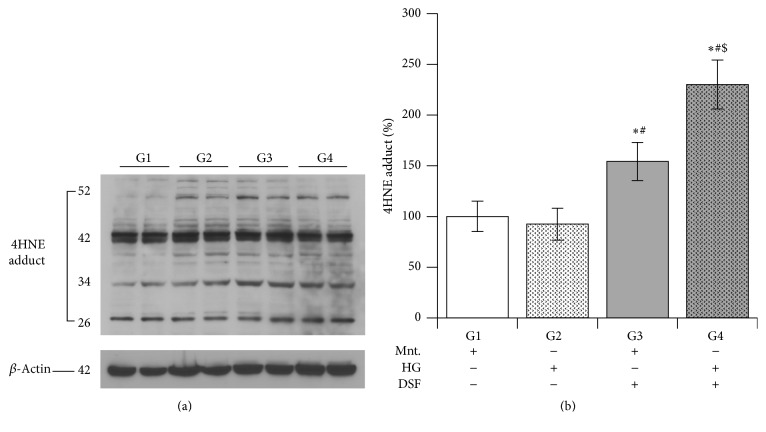
Disulfiram (DSF) pretreatment and 4HNE protein adducts. (a) Western blot images of 4HNE adducts were shown. Beta-actin was used as a loading control. (b) The quantification data of Western immunoblot. Disulfiram (DSF) (10 *μ*M) treatment increases 4HNE protein adduct formation; the data expressed are mean ± SEM. *N* = 4–6. G1, G2, G3, and G4 depict mannitol, high glucose stress, DSF + mannitol, and DSF + high glucose stress, respectively. ^*∗*^ *p* < 0.05 versus G1, ^#^ *p* < 0.05 versus G2, and ^$^ *p* < 0.05 versus G3.

DSF pretreatment for 2 hours significantly augmented 4HNE adducts formation in cells subjected to high glucose stress compared to DSF-untreated cells presented with equimolar concentrations of glucose or mannitol in their culture medium. Moreover, among the DSF-pretreated cells, increase in 4HNE adduct formation was found at a significantly higher amount in the HG group compared to the Ctrl group (Figure 3).

### 3.4. Effect of DSF Treatment on Cell Death in Cultured H9C2 Cardiomyocytes Subjected to High Glucose Stress

**Figure 4:**
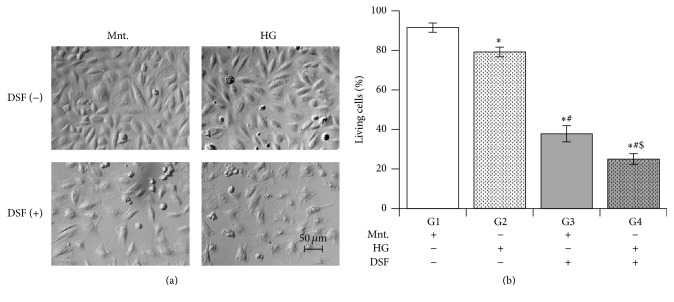
Disulfiram (DSF) pretreatment and cell death. (a) Representative Hoffman modulation-contrast microscopic images of cells were shown. The normal cell morphology was maintained in mannitol with no DSF. However, the cells from all other 3 groups were depicting cell death (shape altered with cell damage) from all 4 groups in various degrees. In the HG and DSF group, lot of cells show cell disintegration depicting cell death. (b) DSF 10 *μ*M treatment leads to cell death cultured H9C2 cardiomyocytes subjected to high glucose stress (HG) compared to equimolar mannitol (Ctrl). Trypan exclusion assay was used to measure the cell death. The data expressed are mean ± SEM. *N* = 4–6. G1, G2, G3, and G4 depict mannitol, high glucose stress, DSF + mannitol, and DSF + high glucose stress, respectively. ^*∗*^ *p* < 0.05 versus G1, ^#^ *p* < 0.05 versus G2, and ^$^ *p* < 0.05 versus G3.

High glucose stress induced cell death in cultured H9C2 cardiomyocytes. Pretreatment with DSF significantly increased cell death in the high glucose group compared to the untreated high glucose group. Surprisingly, DSF pretreatment significantly increased cell death in the mannitol group compared to cells in the DSF-untreated group. Among the DSF treated groups, cell death was significantly increased in the HG group compared to the mannitol group (Figure 4).

### 3.5. Effect of DSF Treatment on Mitochondrial Membrane Potential in Cultured H9C2 Cardiomyocytes Subjected to High Glucose Stress

**Figure 5:**
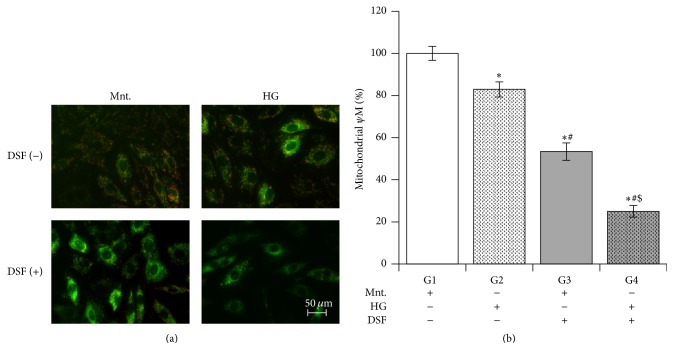
Disulfiram (DSF) pretreatment and mitochondrial membrane potential. (a) Representative microscopic images of cells with JC-1 dye to measure mitochondrial membrane potential (Ψ). Increase in red to green florescence ratio indicates high membrane potential as shown in mannitol group, while increased green florescence indicates drop in mitochondrial Ψ. (b) DSF 10 *μ*M treatment reduces mitochondrial Ψ when measured using JC-1 dye in cultured H9C2 cardiomyocytes subjected to high glucose stress (HG) compared to equimolar mannitol (Ctrl). The data expressed are mean ± SEM. *N* = 4–6. G1, G2, G3, and G4 depict mannitol, high glucose stress, DSF + mannitol, and DSF + high glucose stress, respectively. ^*∗*^ *p* < 0.05 versus G1, ^#^ *p* < 0.05 versus G2, and ^$^ *p* < 0.05 versus G3.

Pretreatment with DSF reduced mitochondrial membrane potential in the HG group compared to the DSF-untreated HG and Ctrl groups. Furthermore, DSF enhanced the reduction in mitochondrial membrane potential in cultured H9C2 cardiomyocytes subjected to high glucose stress compared to cells presented with equimolar mannitol in their culture medium (Figure 5).

HG increased mitochondrial ROS in cultured H9C2 cardiomyocytes which was further enhanced in both Ctrl and HG groups with DSF pretreatment (Supplementary Figure  2).

### 3.6. Effect of DSF Treatment on Mitochondrial Respiration in Cultured H9C2 Cardiomyocytes Subjected to High Glucose Stress

**Figure 6:**
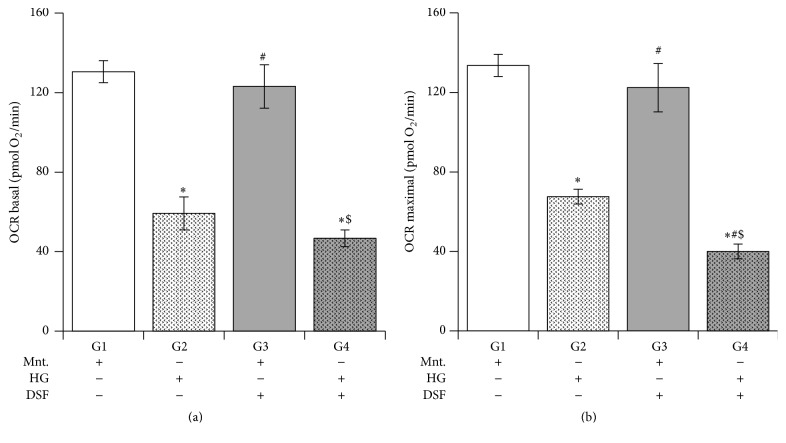
Disulfiram (DSF) pretreatment and mitochondrial oxygen consumption rate (OCR). (a) Basal mitochondrial OCR from G1, G2, G3, and G4 which depict mannitol, high glucose stress, DSF + mannitol, and DSF + high glucose stress. *N* = 4–6. (b) Maximal mitochondrial OCR with FCCP treatment. G1, G2, G3, and G4 depict mannitol, high glucose stress, DSF + mannitol, and DSF + high glucose stress, respectively. *N* = 4–6. ^*∗*^ *p* < 0.05 versus G1, ^#^ *p* < 0.05 versus G2, and ^$^ *p* < 0.05 versus G3.

The mitochondrial OCR was measured to determine mitochondrial respiration. Basal and FCCP-induced maximal OCR were decreased in HG group compared to Ctrl group. Pretreatment with DSF enhanced maximal OCR reduction significantly in HG group but not in Ctrl group (Figure 6).

## 4. Discussion

In this study, we report that the pharmacological inhibition of ALDH2 potentiates high glucose induced deleterious effects in cultured H9C2 cardiomyocyte cell lines.

In a recent study, we reported that ALDH2 inhibition by 75 *μ*M DSF* per se* attenuated mitochondrial respiration and induced cell death in cultured H9C2 cardiomyocyte cell lines without any stress [15]. While DSF inhibited recombinant mitochondrial ALDH2 enzyme with an IC_50_ value of 36.4 *μ*M [23], we found that DSF 75 *μ*M actually attenuated 50% of ALDH2 activity in H9C2 cells without any stress [15]. However the treatment duration was only 2–4 hours without any stress. In this study, we plan to pretreat cells with DSF in high glucose stress. In order to do this, we performed a pilot study to determine the DSF dose as explained in the method section. We first inhibited ALDH2 activity in H9C2 cells using DSF and then subjected them to high glucose stress. To induce high glucose stress* in vitro* the cell culture medium was replaced with 33 mM D-glucose or 33 mM D-mannitol as per previous reports [24]. As we explained in our previous report [15], H9C2 cardiomyocyte cell lines are suitable alternatives for primary cardiomyocytes to be used in hyperglycemic studies. In fact, it was shown that isolated cardiomyocytes and H9C2 cells respond similarly under high glucose stress [25]. Using H9C2 cells was a choice that is logically supported.

We and others reported that hyperglycemia decreases myocardial ALDH2 activity in rodents [19, 26, 27]. The possible mechanism that we propose for this reduction is that hyperglycemia increases ROS generation which attenuates ALDH2 activity and thereby increases 4HNE adduct formation, which ultimately leads to cellular dysfunction and then death. Increased ROS in type 1 and type 2 diabetes is implicated in the development of diabetic cardiomyopathy [28, 29].

DSF pretreatment significantly increased ROS levels in H9C2 cells with mannitol. This result suggests that ALDH2 inhibition by DSF itself can induce ROS generation independent of high glucose stress. DSF treatment further enhanced the increase in ROS levels in high glucose stress compared to control. DSF pretreatment reduced ALDH2 activity more in cells with high glucose compared to control. It appears DSF pretreatment made the cells more susceptible to high glucose mediated ALDH2 impairment. Next, we found a significant increase in 4HNE protein adducts only in DSF-pretreated cells with high glucose stress. This increase was not present in the control group or the DSF-untreated HG group. Though we observed increased ROS levels and reduced ALDH2 activity in the control group with DSF treatment, we did not observe an increase in the level of 4HNE protein adducts. The exact mechanism for this discrepancy is unknown. However we suppose the 20% ALDH2 activity remaining may be sufficient to metabolize 4HNE in the control group. Perhaps, this could have caused the observed reduction in the level of 4HNE protein adducts [30].

The mitochondrial membrane potential was reduced in the DSF treated HG group compared to the DSF-untreated HG group and DSF treated control group. It unequivocally suggests that DSF increases the susceptibility in cells subjected to high glucose stress to a drop in mitochondrial membrane potential despite the difference in glucose levels. Progressive decrease in mitochondrial transmembrane potential is associated with apoptotic cell death [31]. In our study, there was a decrease in the mitochondrial potential under the hyperglycemic condition, suggesting that hyperglycemia could act as an apoptosis-inducing stimulus. Therefore, an intact transmembrane and its potential are indispensable for normal mitochondrial function.

Next, we found mitochondrial OCR at basal and FCCP-induced maximal response were reduced in HG stress. DSF further augmented the reduction in OCR. As per our earlier report, ALDH2 inhibition* per se* attenuated OCR and further this reduction in OCR is linked with cell death [15].

High glucose alone caused 20% cell death. But DSF pretreatment increased the cell death to more than 90% in the high glucose group. Surprisingly, DSF increased cell death to 80% in control cells as well. This data again implicates the role of ALDH2 inhibition in maintaining cell viability and potentiating high glucose mediated cell death. This was also seen in our earlier study as ALDH2 inhibition by DSF and ALDH2 siRNA contributed to cell death by decreasing mitochondrial function in H9C2 cells during 2–4 hours [15]. Decrease in ALDH2 activity by DSF without causing 4HNE adducts formation itself was noted to cause significant reduction in mitochondrial reserve capacity and subsequently cell death in H9C2 cells [15]. Similarly we observed an increase in cell death in control cells perhaps due to the reduction in mitochondrial respiratory defects demonstrated previously [15]. Strikingly, the ALDH2 inhibition by DSF led to a significant increase in 4HNE adducts as well as a decrease in mitochondrial membrane potential in cells under high glucose stress. Earlier reports have shown that hyperglycemia reduced the activity and levels of myocardial ALDH2 [27, 32]. Overexpression of ALDH2 conferred cardioprotection by attenuating 4HNE toxicity in STZ-injected hyperglycemic mice [32]. Taken altogether, we suggest ALDH2 activity plays an important role in the process of hyperglycemia-induced cardiac damage. Similarly we speculate that the hyperglycemic stress in ALDH2^*∗*^2 carriers with diabetes or noncarriers with other comorbidities with low ALDH2 activity may be vulnerable to cardiac tissue damage. Our previous observation points out that long-term diabetes in patients reduces ALDH2 activity and increases 4HNE adduct formation in diabetic hearts [26]. It also implies that chronic hyperglycemia can lead to an inevitable decrease in ALDH2 activity and cause an exacerbation of hyperglycemia-induced cardiac damage.

We conclude this report by stating that the inhibition of ALDH2 activity potentiates high glucose stress-induced deleterious effects in cardiac cells. Our study implicates individuals with compromised ALDH2 activity may have higher propensity to develop diabetic cardiac complications or increase the severity of the damage.

## Supplementary Material


Supplementary Figure  1: Dose-response curve of disulfiram with ALDH2 activity in H9c2 cells. H9c2 cells were exposed to difference concentration of DSF (0–10 μM) over night. The inhibition of ALDH2 activity presents a DSF dose-dependent manner. Supplementary Figure  2: ALDH2 inhibition by pretreating with disulfiram (DSF) and superoxide levels in mitochondria. (a) Representative photomicrographs of MitoSOX staining from each treatment groups. The red fluoresce indicates superoxide in mitochondria. (b) Increase superoxide in mitochondria in cultured H9C2 cardiomyocytes subjected to high glucose stress (G2) compared to equimolar mannitol (G1). Disulfiram (DSF) pretreatment increased ROS levels in both mannitol (G3) and high glucose (G4) groups. The data expressed are mean ± SEM. *N* = 4–6. ^*∗*^ *p* < 0.05 versus G1, ^#^ *p* < 0.05 versus G2, and ^$^ *p* < 0.05 versus G3.
