# Supplementary material for: ALDH2 Inhibition Potentiates High Glucose Stress-Induced Injury in Cultured Cardiomyocytes
Source: J Diabetes Res. 2016 Nov 2;2016:1390861. doi: 10.1155/2016/1390861 (PMC5110883; doi:10.1155/2016/1390861)
Supplement: Supplementary file 1 — Supplementary Figure 1: Dose-response curve of disulfiram with ALDH2 activity in H9c2 cells. H9c2 cells were exposed to difference concentration of DSF (0–10 μM) over night. The inhibition of ALDH2 activity presents a DSF dose-dependent manner. Supplementary Figure 2: ALDH2 inhibition by pretreating with disulfiram (DSF) and superoxide levels in mitochondria. (a) Representative photomicrographs of MitoSOX staining from each treatment groups. The red fluoresce indicates superoxide in mitochondria. (b) Increase superoxide in mitochondria in cultured H9C2 cardiomyocytes subjected to high glucose stress (G2) compared to equimolar mannitol (G1). Disulfiram (DSF) pretreatment increased ROS levels in both mannitol (G3) and high glucose (G4) groups. The data expressed are mean ± SEM. N = 4–6. ∗ p < 0.05 versus G1, # p < 0.05 versus G2, and $ p < 0.05 versus G3. [file 1390861.f1.pdf]

Supplementary

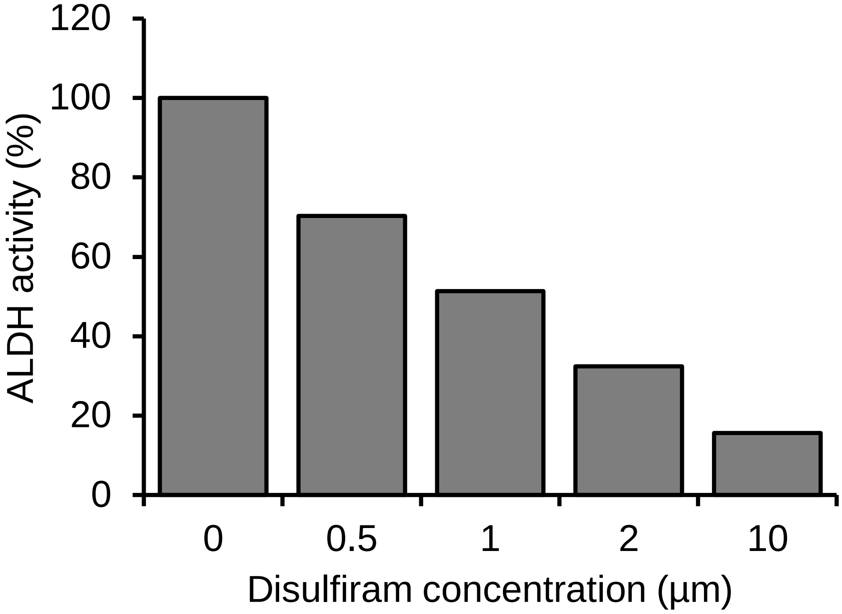

Supplementary figure 1: Dose-response curve of disulfiram with ALDH2 activity in H9c2 cells.

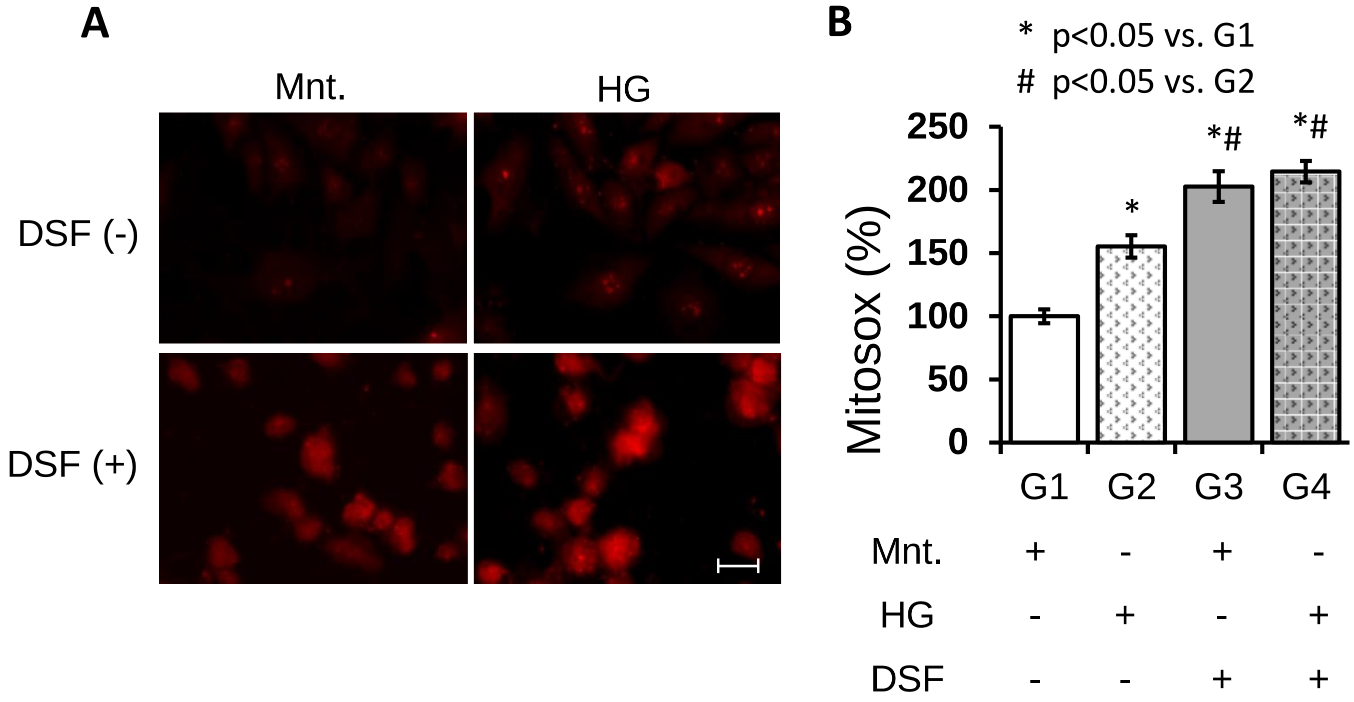

Supplementary figure 2: A. Representative photomicrographs of MitoSOX staining from each treatment groups. B. The quantification of mitochondrial ROS levels in H9c2 cells stained with MitoSOX.
